# Treatment of worry and comorbid symptoms within depression, anxiety, and insomnia with a group-based rumination-focused cognitive-behaviour therapy in a primary health care setting: a randomised controlled trial

**DOI:** 10.3389/fpsyg.2023.1196945

**Published:** 2023-09-07

**Authors:** Daniel Wallsten, Annika Norell, Malin Anniko, Oskar Eriksson, Varja Lamourín, Ida Halldin, Tina Kindbom, Hugo Hesser, Edward Watkins, Maria Tillfors

**Affiliations:** ^1^Department of Social and Psychological Studies, Karlstad University, Karlstad, Sweden; ^2^Faculty of Health and Science, Kristianstad University, Kristianstad, Sweden; ^3^School of Behavioral, Social and Legal Sciences, Örebro University, Örebro, Sweden; ^4^Kronoparken Primary Healthcare Center, Karlstad, Sweden; ^5^Department of Behavioral Sciences and Learning, Linköping University, Linköping, Sweden; ^6^Mood Disorders Centre, School of Psychology, University of Exeter, Exeter, United Kingdom

**Keywords:** anxiety, depression, group therapy, insomnia, repetitive negative thinking, rumination-focused CBT

## Abstract

**Introduction:**

Repetitive negative thinking (RNT) has been described as a maintaining transdiagnostic factor for psychopathology within the areas of depression, anxiety and insomnia. We investigated the effects of rumination-focused cognitive-behaviour therapy (RF-CBT) in a group format at a primary health care centre on symptoms of depression, anxiety, insomnia, RNT, and quality of life. The participants presented clinical symptom levels of worry and at least two disorders among anxiety disorders, major depressive disorder, and insomnia disorder.

**Methods:**

A randomised controlled superiority parallel arm trial was used. 73 participants were included and randomised in pairs to either group-administered RF-CBT or a waiting list condition. The primary outcomes were self-rated worry and transdiagnostic symptoms (depression, anxiety, and insomnia). Intention-to-treat analyses of group differences were conducted using linear mixed models. Adverse side effects and incidents were presented descriptively.

**Results:**

Group RF-CBT significantly reduced self-reported insomnia at post-treatment and self-reported insomnia and depression at the 2 month-follow-up, relative to the wait-list control group. There was no significant difference in change in RNT, anxiety, or quality of life.

**Discussion:**

The current study suggests that group-administered RF-CBT may be effective for insomnia and potentially effective for depression symptomatology. However, the study was underpowered to detect small and moderate effects and the results should therefore be interpreted with caution.

## 1. Introduction

Worry and rumination have been conceptualised as transdiagnostic processes and linked to the onset and maintenance of multiple psychiatric disorders, both separately and together as the concept of repetitive negative thinking (RNT). This includes disorders such as Major Depressive Disorder, Social Anxiety Disorder, Generalised Anxiety Disorder, and Insomnia Disorder (Watts et al., 1994; Harvey et al., 2004; Ehring and Watkins, 2008; Nolen-Hoeksema et al., 2008; Watkins and Roberts, 2020). According to Borkovec et al. (1998), worry has been characterised as” a predominance of verbal thought activity, [that] functions as a type of cognitive avoidance, and inhibits emotional processing.” Similarly, rumination has been described as “a persistent mental attempt at resolving unattained goals, [that] may be initiated by an intrusive concern over a discrepancy between current state and ideal goals” (Martin and Tesser, 2013; Olatunji et al., 2013). Although rumination involves past events whereas worry involves potential future events, RNT captures three overlapping process characteristics: (1) repetitive, passive and/or (2) relatively uncontrollable (i.e., perceived as difficult to inhibit or withstand from) and (3) focused on negative content. Rumination has been associated with depression, whereas worry have been more broadly associated with anxiety disorders and insomnia (Ehring and Watkins, 2008; Olatunji et al., 2010; Spinhoven et al., 2018). However, these perhaps intuitive associations have also been questioned. For example, Hoyer et al. (2009) found, although in a limited non-clinical sample, that worry was a better predictor for both symptoms of anxiety and emotional symptoms, than rumination. The authors also concluded that the lay term anxiety was strongly associated with *The Penn State Worry Questionnaire* (PSWQ; Meyer et al., 1990) and that the PSWQ therefore can be considered face valid for screening people with high levels of worry and thus suitable for clinical settings. According to Spinhoven et al., RNT have been operationalized with different measures within worry and rumination, which typically are highly correlated, but also through measures that focus directly on the common variance. A meta-analysis from 2018 suggested that rumination-focused cognitive-behaviour therapy (RF-CBT) may be more efficacious than other approaches in addressing RNT (Spinhoven et al., 2018). RF-CBT was originally developed to address depressive rumination (Watkins et al., 2007) and has demonstrated promising results in randomised clinical trials in both individual therapies and group formats (Watkins et al., 2007, 2011; Topper et al., 2017; Hvenegaard et al., 2020). RF-CBT addresses RNT with both common techniques associated with functional analysis, self-compassion, values, and mindfulness, but also specifically through the participants process-style as they get to practise distinguishing concrete or constructive thinking from abstract and unconstructive thinking.

Despite the promising results of RF-CBT and the previously emphasised theoretical link between RNT and psychiatric disorders, there has been limited research on the effects of RF-CBT on disorders other than depression. In an explorative clinical trial in adolescents, the effects of RF-CBT on anxiety, with behavioural activation and global functioning as secondary outcomes, were investigated. Significant results were found regarding decreased anxiety and increased behavioural activation but not for improved global functioning in an adolescent sample (Feldhaus et al., 2020). Another study investigated the prevention of anxiety disorders and depression in a randomised controlled trial by addressing RNT in a sample of adolescents and young adults with elevated worry and rumination (Topper et al., 2017). The authors found that both group-and online RF-CBT significantly reduced the onset of depression and anxiety disorders over the subsequent 12 months. Even though RNT has been linked to sleep problems (Harvey et al., 2004; Ehring and Watkins, 2008), to our knowledge, there have been no studies on the effects of RF-CBT on insomnia disorder. Sleep problems are also assumed to interfere with psychological treatment as for example both concentration and emotions regulation are affected by deprived sleep (Walker and van der Helm, 2009; Lim and Dinges, 2010). This points to the potential benefits of RF-CBT for people with comorbid Insomnia Disorder and therefore also to the need of investigating how RF-CBT may affect comorbid problems including Insomnia Disorder. Further, insomnia have been identified as a maintaining factor of depression, and a risk factor for new depressive episodes (Riemann et al., 2020). Clinical trials in which depression is addressed should therefore investigate to what extent symptoms of insomnia may be affected by the treatment, as this might shed a light on whether a salient risk factor for new episodes has been successfully targeted. To summarize, although being theoretically motivated, there is currently little evidence on how RF-CBT may be effective for problems besides those associated with depression. There are significant gaps concerning the knowledge on efficacy and effectiveness of RF-CBT on disorders within anxiety disorders and insomnia.

Furthermore, to our knowledge, no clinical trials have yet evaluated the effects of RF-CBT on a deliberately recruited transdiagnostic adult sample with clinical symptom levels among the common areas of Major Depressive Disorder, anxiety disorders (Specific Phobia, Social Anxiety Disorder, Panic Disorder, Agoraphobia, Generalized Anxiety Disorder, Separation Anxiety Disorder) and Insomnia Disorder, as defined by the Diagnostic and Statistical Manual of Mental Disorders (American Psychiatric Association, 2013). Also, no identified trials were conducted in a primary health care setting, where about 30% of patients meet the criteria for at least two comorbid psychiatric disorders (Kessler et al., 2005; Roca et al., 2009). Even if evidence-based treatment protocols for individual disorders already exist, a transdiagnostic treatment that addresses a potential maintenance factor seen in multiple disorders could enhance treatment effects among participants with elevated levels of that factor. Thus, even with limited data, the possibility of a transdiagnostic approach addressing persistent negative thinking to treat multiple disorders appears plausible. Finally, few studies on RNT have up and until now focused on worry rather than depressive rumination (Spinhoven et al., 2018).

Therefore, the current study aimed to investigate the effectiveness of group-delivered RF-CBT with a randomised controlled trial in a sample of participants with clinical symptom levels of worry and at least two disorders among anxiety disorders, Major Depressive Disorder, and Insomnia Disorder. We hypothesised that participants randomised to RF-CBT would report greater reductions in anxiety, insomnia, depression, and worry, and greater increases in perceived quality of life from baseline to each of the following time points (post, FU1, FU2) than those randomised to a wait-list control group. We also explored if participation in treatment was associated with any adverse side-effects or events.

## 2. Method

### 2.1. Research design

The design was a randomised controlled superiority parallel arm trial with 73 participants who were randomised 1:1 into two conditions: treatment (group RF-CBT; *n* = 36) and wait-list control group (WL; *n* = 37). Measurements were taken before, immediately after, and 2 and 6 months after the end of the treatment. During measurements, participants randomly assigned to the wait-list control group completed the same evaluations as those participating in the treatment group at the same time points, except for the 6-months follow-up since the participants in the waitlist-control group received their treatment after the 2-month follow-up measurements.

### 2.2. Participants

An ethical approval was obtained from the regional ethics review board in Uppsala, Sweden (Dnr: 2018/197). All participants received written information concerning the study and their participation, and they completed an informed consent form. All data were coded, and the clinical trial was registered at: https://www.anzctr.org.au, Clinical registration number: ACTRN12618001614280.

**Figure 1:**
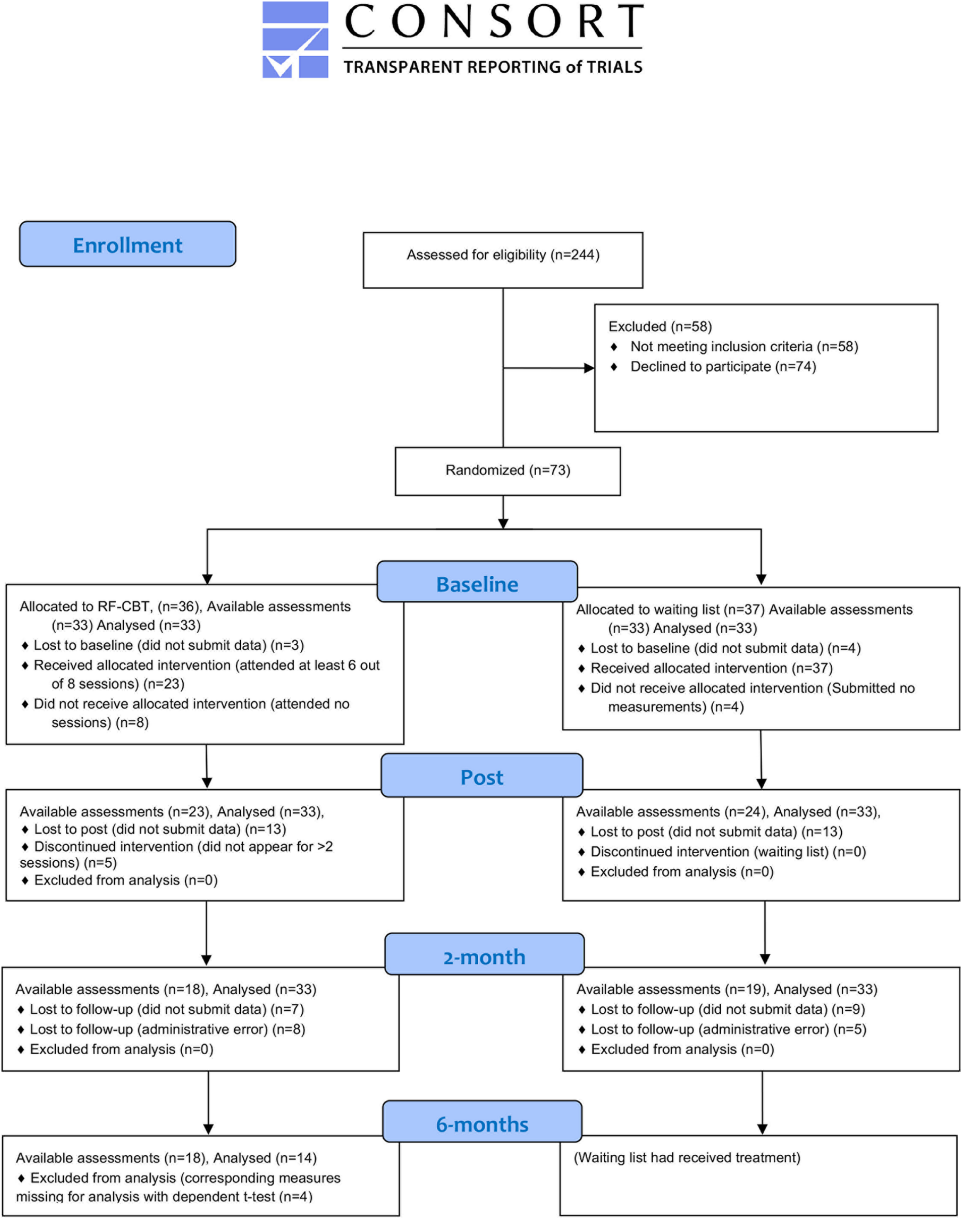
Flow diagram of participants (Note that linear mixed models use all available data points and may result in more analyses than available assessments because of how missing data are treated).

Participants (*n* = 73) were recruited through advertisements and articles in regional media, regional radio information, social media ads, and information provided in local primary health care centres, between September 2018 and April 2019. The flow of participants through the study is depicted in Figure 1. Applicants were directed to a web page with information about the study and a secure digital platform to collect research data to convey their interest and initial information for the screening procedure.

### 2.3. Screening and assessment process

In this study, comorbid problems (clinical symptom levels of worry and at least two disorders among anxiety disorders, major depressive disorder, and insomnia disorder) was defined as either reporting clinicals symptom levels of worry and meeting criteria for two disorders among major depressive disorder, insomnia disorder or any anxiety disorder as defined by the diagnostic manual of mental disorders 5 (DSM-5; American Psychiatric Association, 2013) according to the Mini-International Neuropsychiatric Interview (M.I.N.I.; Sheehan et al., 1998) and Insomnia Disorder according to The Duke Structured Interview for Sleeping Disorders (DSISD; Edinger et al., unpublished material; Taylor et al., 2018), OR reporting clinicals symptom levels of worry and meeting criteria for one disorder according to the M.I.N.I. or the DSISD, AND report clinical symptoms levels within at least one of the areas depression, anxiety or insomnia disorder, with an established self-rating scale. Clinical symptom levels of worry were defined in line with the cut-off scores for general anxiety disorder, thus a total score of ≥45^1^ on the Penn State Worry Questionnaire (PSWQ; Meyer et al., 1990; Behar et al., 2003), thus the closest to a psychometric operationalization of clinical worry that could be identified.

The screening procedure was conducted in two steps. *First*, participants were deemed potentially eligible for the study if they had conveyed interest in participation, were ≥18 years old, and provided informed consent and health information. They had to report established clinical levels of worry and on two out of three of the following self-rating scales: Major Depressive Disorder [total score of ≥13 on the Montgomery-Åsberg Depression Rating Scale (MADRS-S; Svanborg and Asberg, 1994)], Insomnia Disorder [total score of ≥8 on the Insomnia Severity Index (ISI; Bastien, 2001], Anxiety [total score of ≥8 on the Overall Anxiety Severity and Impairment Scale (OASIS; Norman et al., 2006)]. If any pharmacological treatment for anxiety, depression, or insomnia disorder occurred, the dose had to have been stable over the past 2 months or longer. *Second*, those who met the initial criteria were contacted by the research group for further assessment over the telephone, including the clinical interviews M.I.N.I. (Sheehan et al., 1998) and DSISD (Edinger et al., unpublished material; Taylor et al., 2018). The included participants had to meet the criteria for comorbid problems as defined above.

The following conditions were cause for *exclusion*: Severe depression (total score of ≥30^2^ on MADRS-S), ongoing psychosis or mania (M.I.N.I.), suicidal tendencies (total score of ≥4 on MADRS-S item 9), or other concurrent psychological treatments. Those who initially reported severe depression, or an elevated risk of suicide were contacted by licenced psychologists for further advice. The M.I.N.I. and the DSISD were conducted by licensed clinical psychologists and students enrolled in the clinical psychologist master’s program under supervision by a licensed clinical psychologist. All interviewers received training in DSISD and a test screening with an actor to ensure interrater reliability. There was full agreement between interviewers concerning diagnoses and recommendations for participation. All interviewers had previous experience from conducting the M.I.N.I., and the master students also needed to consult their assessments with a licenced psychologist within the research group.

Applicants were then contacted by the research group and either offered participation or advice on self-help literature and where to apply for sufficient health care. Each included participant was provided with information about the randomisation procedure, the baseline measurements, and the estimated start date for the treatment group. After the inclusion, each participant received an email with a link to the secure research data collection platform through which the baseline measurements were administered.

### 2.4. Randomisation

For every two enrolled participants, a 1:1 block randomisation was conducted to ensure equal group sizes.^3^ The randomisation was made by an independent researcher at Karlstad University with the Microsoft excel Rand function. When each participant had provided baseline measurement data, they were phoned by a research group member and received practical information about their assignment. The participants who received group-administered RF-CBT visited a local primary healthcare centre once every week for eight consecutive weeks, 2 h at a time. The time between indication of interest, screening, enrolment, allocation, and the beginning of treatment within the treatment condition varied between the participants depending on the time point of their application but was kept at a minimum duration. At most, about 2 months between application and treatment start, and about 1 month between enrolment and the beginning of treatment.

### 2.5. Measures

All regular measurements with self-rating scales (at baseline = 0 weeks, post = 10 weeks,^4^ follow-up 1 = 2-months and follow-up 2 = 6 months^5^) were sent by email through which participants could access a secure digital system for collecting research data, between November 2018 and December 2019. Three reminders were sent after 2, 4, and 6 days. The baseline measurements were administered up to 2 weeks before the treatment. After the first session, the Credibility and Expectancy Questionnaire (CEQ; Devilly and Borkovec, 2000) was provided to assess perceived credibility and expectancy. After the last session, the Negative Effects Questionnaire (NEQ-20; Rozental et al., 2016) was provided to assess adverse side effects and incidents. All self-report data except the NEQ and the CEQ were collected digitally and ensured that no individual items were missing at any measurements.

#### 2.5.1. Primary outcome measures

##### 2.5.1.1. Worry

*The Penn State Worry Questionnaire* (PSWQ; Meyer et al., 1990) was used to assess the severity of worry. The PSWQ consists of 16 items, with each item assessing the extent of worry (e.g., “My worries overwhelm me” or “Once I start worrying, I cannot stop”) rated on a Likert-scale from 1 (i.e., “not at all typical of me”) to 5 (i.e., “very typical of me”), rendering a total score between 16 and 80. A total score of ≥45 was used to indicate clinical levels of worry (Meyer et al., 1990). Cronbach’s α in the present study at baseline was 0.86.

##### 2.5.1.2. Transdiagnostic symptoms

*The Insomnia Severity Index* (ISI; Bastien, 2001) was used to assess symptoms of insomnia disorder. The ISI consists of seven items (e.g., “How difficult is it for you to fall asleep?”) rated on a scale from 0 (i.e., “not at all”) to 4 (i.e., “much”), rendering a total score between 0 and 28. A total score of ≥8 was used to indicate clinical levels of insomnia symptoms. Cronbach’s α in the present study at baseline was 0.85.

*The Montgomery-Åsberg Depression Rating Scale* (MADRS-S; Svanborg and Asberg, 1994) assessed depressive symptoms. The MADRS-S consists of nine items (e.g., “Here you should assess your interest in your surroundings, in other people, and in activities that normally give you pleasure”) rated on a scale between 0 (i.e., description of mild or absent symptoms) to 6 (i.e., description of severe symptoms), rendering a final score between 0 and 45. A total score of ≥13 was used to indicate clinical levels of depression. A score of ≥30 (the threshold was later corrected to ≥34) indicated severe depression. Reported scores of ≥4 on item 9 indicate elevated risks of suicide. Cronbach’s α in the present study at baseline was 0.85.

*The Overall Anxiety Severity and Impairment Scale* (OASIS; Norman et al., 2006) was used to assess anxiety symptoms. The OASIS consists of five items (e.g., “In the past week, how often have you felt anxious?”) rated on a scale from 0 (i.e., description of low severity/frequency of symptoms) to 4 (i.e., description of high severity/frequency of symptoms), rendering a final score between 0 and 20. A total score of ≥8 was used to indicate clinical anxiety levels (Campbell-Sills et al., 2009). Cronbach’s α in the present study at baseline was 0.87.

#### 2.5.2. Secondary outcome measures

*The Perseverative Thinking Questionnaire* (PTQ; Ehring et al., 2011) was used to assess the severity of RNT. The PTQ consists of 15 items (e.g., “the same thoughts keep going through my mind again and again) rated on a scale from 0 (i.e., “never”) to 4 (i.e., “almost always”), rendering a final score between 0 and 60. No clinical cut-offs are available for PTQ. However, higher scores indicate more severe problems. Cronbach’s α in the present study at baseline was 0.93.

*Brunnsviken Brief Quality of life scale* (BBQ; Lindner et al., 2016) was used to assess quality of life and consists of 12 items (e.g., “I am satisfied with my leisure time: I have the opportunity to do what I want to relax and enjoy myself”) rated on a Likert-scale between 0 (i.e., “do not agree at all”) and 4 (i.e., “agree”), rendering a final score between 0 and 48. No cut-offs are available for BBQ. However, lower scores indicate lower quality of life. Satisfactory psychometric properties have been reported concerning the validity, internal consistency, and reliability (Lindner et al., 2016). Cronbach’s α in the present study was calculated to be 0.79.

*The Negative Effects Questionnaire* (NEQ-20; Rozental et al., 2016) was used to measure adverse side effects and incidents post-treatment and consisted of 20 questions (e.g., “I started feeling ashamed in front of other people because I was having treatment” and is rated in three steps. The first step indicates whether a phenomenon occurred during treatment or not with yes and no questions. The second step rates the severity of the effect on a five-point scale ranging from “not at all to “extremely.” The third step of each question states the cause of the effect; “The treatment I received” or “Other circumstances.” Cronbach’s α was not calculated in the present study due to large amounts of missing data (see the result sections).

#### 2.5.3. Other measures

The Credibility and Expectancy Questionnaire (CEQ; Devilly and Borkovec, 2000) was used to assess perceived treatment credibility after the first session and consisted of six items (e.g., “at this point, how much sense does the therapy offered to you make?”) rated between 1 (i.e., “not at all”) and 9 (i.e., “very much”; item 1–3 and 5) or 0 to 100% (item 4 and 6). The first three items have constituted a cognitive factor (credibility), whereas the last three items have constituted an affective factor (expectancy) concerning the treatment. To handle the different scales within CEQ, individual composite scores were calculated by first standardising each item and then summing the items included in each factor case wise (Devilly and Borkovec, 2000). Mean composite scores were presented for items 1–3 and item 4 according to Thompson-Hollands et al. (2014). Cronbach’s α in the present study was calculated to be 0.73 for factor 1 and to 0.68 for factor 2.

#### 2.5.4. Structured interviews

*The Duke Structured Interview for Sleep Disorders* (DSISD; Edinger et al., unpublished material; Taylor et al., 2018) was used to assess DSM-5 criteria for insomnia disorder and sleep apnea during the screening procedure. Satisfactory psychometric properties have been demonstrated concerning discrimination between disorders, inter-rater reliability, and reliability (Carney et al., 2009; Taylor et al., 2018).

*The Mini-International Neuropsychiatric Interview* (MINI; Sheehan et al., 1998) covered DSM-5 criteria for 17 different psychological disorders and was used to assess comorbid disorders for inclusion and exclusion symptoms present study. Section A, B, C, D, E, F, K, N, and O were used during the screening procedure. Satisfactory psychometric properties have been demonstrated concerning the validity of all diagnostic areas except drug dependence and test–retest-and inter-rater reliability (Sheehan et al., 1998).

### 2.6. Treatment

The treatment protocol used in the study was based on the published manuals “Rumination-Focused Cognitive-Behavioral Therapy for Depression” (Watkins, 2018) and “Ruminationsfokuseret Kognitiv Adfærdsterapi for Depression” (Møller et al., 2017). All material was translated to Swedish and modified to fit eight 2-h group sessions with nine participants (including 15-min breaks). The treatment modules are presented in Table 1). All sessions included basic therapeutic techniques, such as normalisation and validation, and general principles for cognitive-behavioural therapy, such as presenting an agenda, psychoeducation, within-session practice and home assignments. Functional analysis and “if-then plans” were used throughout the treatment. The “If-then plans” link with the functional analysis of RNT and entails finding the triggers and warning signs for RNT and then making plans for alternative constructive strategies to do instead of RNT to those triggers (e.g., trigger = feeling anxious, prior response = abstract rumination around “What if I’m not good enough? Why is this hard?,” alternative strategy = concrete thinking “What steps can I take to prepare?”). For further elaborations and clarifications regarding specific techniques, see Watkins (2018) and Møller et al. (2017). The treatment protocol used in this study may be shared upon on formal request.

**Table 1:** Treatment modules.

Session	Content	Home assignment
1: Emotions, worry and rumination	Practical information, introducing emotions, worry and rumination, functional analysis, worry diary and treatment goal formulation	Complete form for treatment goal formulation and a worry diary
2: Avoidance	Explaining unhelpful avoidance with operant behaviour analysis and if-then plans	Track avoidance with functional analysis and complete forms for If-then plans
3: Relaxation	Functional analysis of worry and rumination (RNT) and introducing relaxation.	Practice relaxation and if-then plans.
4: Changing process style	Introducing abstract thinking versus concrete and specific thinking	Practice concrete thinking and if-then plans
5: Being present in activities	Introducing mindfulness, flow, and visualising	Practice visualising, being mentally present in activities (“flow”) and if-then plans
6: Self-compassion	Introducing self-compassion	Practice self-compassion and if-then plans
7: Which are your values? Learning from experience	Introducing values, relapse prevention and maintenance plan	Complete plan for maintenance and relapse prevention
8: Ending and evaluation of treatment goals	Follow-up treatment goals, evaluating treatment	

### 2.7. Therapists

There were four RF-CBT groups run for the intervention arm, each delivered by two group leaders, of which at least one was an experienced licensed psychologist with training in CBT. Eight therapists in total delivered the four RF-CBT groups. Four RF-CBT groups were run for the wait-list control group at 8 weeks. The treatment protocol was administered by two group leaders, of which at least one was a licensed psychologist. The therapists were either licensed psychologists, graduates in clinical psychology, or final year undergraduate students in psychology who had received clinical training. All therapists were involved in the formulation of the treatment protocol, conducted extensive self-studies of RF-CBT, and attended discussion seminars with the project group.

### 2.8. Adherence to treatment and dropout

Data on attendance were collected for all sessions, but no measure for in-session adherence or homework assignments was used. Because of ethical regulations, participants did not have to state the reason for their dropout or missed sessions, and no systematic investigations for dropouts or missed sessions were made.

#### 2.8.1. Therapist adherence

Two sessions from each of the four treatment groups were randomly selected for external review concerning therapist adherence to the protocol (Sessions 2 and 6 in the first group, sessions 3 and 5 in the second group, sessions 5 and 7 in the third group, session 5 and 6 in the fourth group). A licensed psychologist who was previously involved in developing the treatment protocol listened to complete recordings from the randomly selected sessions. Each element (such as review of previous home assignments, exercises on session theme, review of upcoming homework, evaluation of session) of each session was reviewed. The degree of adherence to each element of the session was evaluated on a scale from 1 to 3, where 1 indicates a low degree of adherence, 2 an acceptable degree, and 3 a full degree of adherence to the protocol.

### 2.9. Power analysis

Power calculations were made for the main statistical analysis Linear mixed models based on mean differences (ES = d = 0.89) in worry (group RF-CBT vs. WL) obtained from Topper et al. (2017). With the desired power of 0.80 alpha level of 0.05, a minimum of 27 participants per arm was required to detect a large standardised mean difference effect size (≥d = 0.80) according to Kazdin and Association, A. P (1992, p.283). In addition, nine participants were added to each arm to handle an expected dropout rate of <20%. During recruitment, an administrative error rendered one extra participant on the waiting list condition, resulting in 73 participants.

### 2.10. Statistical analyses

SPSS was used to analyse data (IBM Corp, 2017; version 26). Continuous variables collected at the primary assessment points (baseline, post-treatment, and 2-month follow-up) were analysed using linear mixed models (covariance pattern models) fitted with restricted maximum likelihood (Hedeker and Gibbons, 2006). Linear mixed models use all available data, account for the correlations between repeated observations, and provide unbiased estimates under a lenient missing data assumption (Hesser, 2015). All individuals with at least one observation on the dependent variable were included in the models, resulting in an intention-to-treat based analysis.^6^ Time was treated as a categorical variable in the model by including two dummy variables representing a change from baseline to post and baseline to follow-up. We analytically determined the best-fitted variance–covariance matrix by comparing various covariance structures to an unstructured form (i.e., full or saturated model for covariance). Estimates of the population’s average differential change over time as a function of treatment conditions were evaluated using the model’s time by condition interaction effects. Based on parameter estimates from linear mixed models we computed effect sizes in the form of standardised mean difference (Cohen’s d) with associated confidence intervals following Feingold’s formulas (Feingold, 2009; Feingold et al., 2014).

The total number of participants within each arm moving across the clinical cut-off levels (PSWQ: 45), ISI: 8, OASIS: 8, MADRS: 13) between base-and post, baseline and 2-month follow-up, and baseline and 6-month follow-up were used to assess clinical significance (Appendix; Table 6).

Item scores on CEQ were standardised and summed in two composites: expectancy (item 1–3) and credibility (item 4–6). Pearson correlations were conducted between the credibility/expectancy composites and change scores on outcome variables with significant effects between baseline and post-treatment measurements and between baseline and the 2-month follow-up measurements. These analyses were conducted to investigate if credibility or expectancy were associated with the treatment progress (Devilly and Borkovec, 2000). Because the averages of standardised items scores equal 0, mean scores and standard deviation for each composite are presented according to Thompson-Hollands et al. (2014).

## 3. Results

### 3.1. Demographic characteristics

In the study, 73 participants between the ages 21 and 75 (M = 47.41 *SD* = 17.23), 54 women and 19 men were included (Table 2).

**Table 2:** Description of the participants.

	RF-CBT		WAITLIST	
	M (SD)	n	M (SD)	n
PSWQ	59.12 (9.81)	33	63.36 (7.98)	33
ISI	15.61 (5.03)	33	15.21 (5.97)	33
OASIS	7.58 (4.1)	33	8.18 (4.05)	33
MADRS-S	18.42 (7.73)	33	20.61 (7.13)	33
BBQ	31.94 (6.75)	33	30.85 (6.94)	33
PTQ	35.03 (10.01)	33	38.09 (8.68)	33
CEQ (credibility)	7.17 (0.94)	28		
CEQ (expectancy)	63.93 (17.07)	28		
Age	51.67 (16.11)	36	43.27 (17.49)	37
Years living in Sweden	50.21 (15.61)	33	43.00 (18.95)	33
Gender		36		37
Female		30		24
Male		6		13
Relationship status				
Married / Living together		18		22
Partner but not living together		1		4
Widow / Widower		3		1
Divorced/separated		5		4
Single		5		2
Other		1		0
Educational level				
Elementary school		0		0
High school/folk high school		10		16
Vocational training		4		2
College / University		19		15
Occupation				
Permanent employment		15		18
Temporary employment		0		1
Unemployed		2		0
Student		0		4
Retired		7		5
Disability pension		0		1
Other		3		2
More than one occupation		6		2
Country of origin				
Sweden		31		30
Other		2		3

PSWQ = Penn state worry questionnaire, ISI = insomnia severity index; OASIS = overall anxiety severity and impairment scale; MADRS-S = Montgomery-Åsberg depression rating scale self-rated; BBQ = Brunnsviken brief quality of life scale; PTQ = perseverative thinking questionnaire; CEQ = credibility/expectancy questionnaire.

### 3.2. Controlled treatment effects at the post-and 2-month follow up

#### 3.2.1. Primary outcomes

Observed means and standard deviations as a function of the condition, along with model-implied effect sizes, are presented in Table 3, and parameter estimates from linear mixed models are presented in Table 4. Observed changes in RF-CBT were all in the expected direction with improvements from baseline to follow-up assessments, and model-implied within-group effects in RF-CBT between baseline and 2-month follow-up were in the range of *d* = 0.65 to *d* = 1.02 for all primary outcomes.

**Table 3:** Observed means and SD for the baseline, post-treatment and 2-month follow-up measurements by group and model-implied between-and within-group effect sizes.

		M *(SD)*			Effect size Cohen’s *d* (95% CI)		
Instrument and condition	Baseline	Posttreatment	2-month follow-up	Within-group, baseline - post-treatment	Within-group, baseline - 2-month follow-up	Between-group, baseline – posttreatment	Between-group, baseline - 2-m FU
PSWQ						0.39 (0.96, −0.18)	0.41 (1.15, −0.33)
Treatment	59.12 (9.81)	53.58 (11.74)	50.03 (10.37)	0.62	1.02		
Waitlist	63.36 (7.98)	61.29 (9.11)	57.94 (8.69)	0.23	0.61		
ISI						0.84 (1.32, 0.36)	0.56 (1.09, 0.03)
Treatment	15.61 (5.03)	11.25 (5.47)	10.21 (5.33)	0.79	0.98		
Waitlist	15.21 (5.97)	15.5 (5.92)	12.9 (5.99)	−0.05	0.42		
OASIS						0.38 (0.86, −0.10)	0.34 (0.86, −0.19)
Treatment	7.58 (4.10)	5.26 (3.73)	4.71 (3.50)	0.57	0.70		
Waitlist	8.18 (4.05)	7.41 (4.74)	6.68 (4.13)	0.19	0.37		
MADRS						0.39 (0.92, −0.14)	0.54 (0.93, 0.14)
Treatment	18.42 (7.73)	13.87 (7.62)	11.78 (6.38)	0.61	0.89		
Waitlist	20.61 (7.13)	18.96 (8.82)	17.95 (6.48)	0.22	0.36		
BBQ						−0.16 (0.25, −0.56)	−0.3 (0.15, −0.74)
Treatment	31.94 (6.75)	34.17 (7.26)	36.35 (5.11)	0.33	0.64		
Waitlist	30.85 (6.94)	32 (6.23)	33.23 (7.77)	0.17	0.35		
PTQ						0.39 (0.78, −0.11)	0.16 (0.69, −0.53)
Treatment	35.03 (10.01)	28.98 (12.15)	28.9 (11.95)	0.65	0.65		
Waitlist	38.09 (8.68)	35.69 (8.57)	33.45 (7.79)	0.26	0.50		

2-m FU = 2-month follow-up measurements PSWQ = Penn state worry questionnaire, ISI = insomnia severity index; OASIS = overall anxiety severity and impairment scale; MADRS-S = Montgomery-Åsberg depression rating scale self-rated; BBQ = Brunnsviken brief quality of life scale; PTQ = perseverative thinking questionnaire.

**Table 4:** Results of the linear mixed-effects regression analyses.

	Linear mixed-effects 95% Cl	
Instrument and condition	Beta (*SE*)	*p*
PSWQ		
Time1 (baseline - posttreatment)	−2.08 (1.79)	0.25
Time2 (baseline - 2-month follow-up)	−5.42 (2.35)	0.02
Tx (group)	−4.24 (2.37)	0.08
Time1 * Tx	−3.47 (2.56)	0.18
Time2 * Tx	−3.67 (3.36)	0.28
ISI		
Time1 (baseline-post)	0.29 (0.93)	0.75
Time2 (baseline-2-month follow-up)	−2.31 (1.02)	0.03
Tx (group)	0.39 (1.39)	0.78
Time1 * Tx	−4.65 (1.33)	<0.01
Time2 * Tx	−3.08 (1.46)	0.04
OASIS		
Time1 (baselinepost)	−0.77 (0.69)	0.27
Time2 (baseline-2-month follow-up)	−1.5 (0.76)	0.05
Tx (group)	−0.61 (1.01)	0.55
Time1 * Tx	−1.54 (0.99)	0.12
Time2 * Tx	−1.37 (1.08)	0.21
MADRS		
Time1 (baselinepost)	−1.64 (1.37)	0.24
Time2 (baseline-2-month follow-up)	−2.66 (1.02)	0.01
Tx (group)	−2.18 (1.83)	0.24
Time1 * Tx	−2.91 (1.97)	0.15
Time2 * Tx	−3.98 (1.47)	0.01
BBQ		
Time1 (baselinepost)	1.15 (0.98)	0.24
Time2 (baseline-2-month follow-up)	2.38 (1.07)	0.03
Tx (group)	1.09 (1.68)	0.52
Time1 * Tx	1.08 (1.4)	0.44
Time2 * Tx	2.02 (1.53)	0.19
PTQ		
Time1 (baselinepost)	−2.4 (1.56)	0.13
Time2 (baseline-2-month follow-up)	−4.64 (2.13)	0.03
Tx (group)	−3.06 (2.42)	0.21
Time1 * Tx	−3.64 (2.24)	0.11
Time2 * Tx	−1.49 (3.06)	0.63

Time1 = baseline – posttreatment, time2 = baseline 2-month follow-up, Tx = treatment variable (1 = treatment, 0 = control). PSWQ = Penn state worry questionnaire, ISI = insomnia severity index; OASIS = overall anxiety severity and impairment scale; MADRS-S = Montgomery-Åsberg depression rating scale self-rated; BBQ = Brunnsviken brief quality of life scale; PTQ = Perseverative thinking questionnaire.

We observed significant time by group interaction effects regarding insomnia symptoms (ISI) between the baseline and post-measurements, with a model-implied between-group effect size of large strength and between baseline and 2-month follow-up measurements with a model-implied between-group effect size of moderate strength, in favour of RF-CBT. A significant time by group interaction effect with a model-implied between-group medium effect size regarding symptoms of depression (MADRS) was found between the baseline and 2-month follow-up measurements in favour of RG-CBT, but no statistically significant time by group interaction between the baseline and post-measurements was detected. No significant time by group interaction effects were found regarding worry (PSWQ) or anxiety symptoms (OASIS).

#### 3.2.2. Secondary outcomes

Concerning secondary outcomes, no significant time by group interaction effects was found for RNT according to PTQ, or quality of life according to BBQ.

### 3.3. Attendance at treatment

Participants’ adherence to treatment is presented in the (Appendix; Table 8).

### 3.4. Follow-up attrition

Out of 73 participants randomised, seven did not report any data at the baseline measurements^7^ (three (8%) in the treatment condition and four (11%) in the wait-list condition). Data from 26 participants were missing at the post-treatment measurements, 13 (36%) in the treatment condition and 13 (35%) in the wait-list control group condition. Data from 36 participants were missing at the 2-month follow-up, 18 (50%) in the treatment condition and 18 (49%) in the wait-list control group condition. Due to an administrative error, nine participants (25%) in the treatment condition and eight (22%) on the waiting list never received their email prompts for the 2-month follow-up. At the 6-month follow up, data from 18 participants (50%) in the treatment condition were collected. Since the participants in the wait-list condition had received treatment at that time point, no comparative data were collected (see further details in Appendix; Table 9).

### 3.5. Therapist’s adherence

The therapist’s adherence to the treatment protocol was reported as high, with some notable decline in ratings concerning the element of post-session evaluation (Appendix; Table 7).

### 3.6. Treatment credibility and expectancy

Twenty-eight out of 36 participants in the RF-CBT condition submitted The Credibility and Expectancy Questionnaire (CEQ) with mean scores of 7.17 (*SD* = 0.94) for credibility and 63.93 (*SD* = 17.07) for expectancy. No significant correlations were found between: credibility and change in ISI scores between the baseline and post measurements, *r(22)* = −0.035, *p* = 0.878 nor between expectancy and change in ISI scores between baseline and post-treatment measurements, *r(22*) = 0.026 *p* = 0.908, nor between credibility and change in ISI scores between baseline and the 2-month follow-up measurements, *r(17)* = −0.299, *p* = 0.244, nor between expectancy and change in ISI scores between the baseline and 2-month follow-up measurements, *r(17)* = 0.043 *p* = 0.870, nor between credibility and change in MADRS-S scores between the baseline and 2-month follow-up measurements, *r(17)* = 0.200, *p* = 0.442, nor between expectancy and change in MADRS-S scores between baseline and the 2-month follow-up measurements, *r(17)* = 0.035 *p* = 0.895. This suggests that neither treatment credibility nor expectancy seems to be associated with the significant effects of RF-CBT on insomnia symptoms (baseline/post, baseline/2-month follow-up) and depression (baseline/2-month follow-up).

### 3.7. Uncontrolled treatment effects over the 6-month follow-up

No statistically significant differences were observed between the 2- and 6-month follow-up measurements for those initially randomised to treatment. This suggests that observed effects on ISI and MADRS-S were maintained throughout the 6-month follow-up (Table 5).

**Table 5:** Within-group-mean-differences in the treatment condition between 2-month and 6-month follow-up measurements.

	2-month follow-up *n* = 14	6-month follow-up *n* = 14	Paired differences	*t*-test			
	M (SD)	M (SD)	M (*SD*)	t	*df*	*p*	Cohen’s d
PSWQ	48.57 (10.27)	46 (8.82)	2.57 (6.24)	1.54	13.00	0.15	−0.27
ISI	10.57 (4.99)	10.07 (4.16)	0.5 (5.03)	0.37	13.00	0.72	−0.11
OASIS	4.43 (2.85)	3.79 (3.09)	0.64 (2.34)	1.03	13.00	0.32	−0.22
MADRS-S	10.86 (5.41)	11.64 (7.03)	−0.79 (5.48)	−0.54	13.00	0.60	0.12
BBQ	36.5 (5.14)	35.86 (5.7)	0.64 (6.08)	0.40	13.00	0.70	−0.12
PTQ	27.14 (12.33)	25.57 (11.06)	1.57 (5.96)	0.99	13.00	0.34	−0.13

PSWQ = Penn state worry questionnaire, ISI = insomnia severity index; OASIS = overall anxiety severity and impairment scale; MADRS-S = Montgomery-Åsberg depression rating scale self-rated; BBQ = Brunnsviken brief quality of life scale; PTQ = perseverative thinking questionnaire. Effect sizes according to Cohen (2013) (0.20–0.49 = small effects, 0.50–0.79 medium effects, 0.80 and above large effects.

### 3.8. Adverse side effects and incidents

A total of 33 participants (including participants from the wait-list control group who were offered treatment after the 2-month follow-up) completed the NEQ-20. A total of 67 instances of adverse side effects were reported by 18 participants, of which 23 were reported as side effects of the treatment rather than other circumstances. A total of 28 instances of reported side effects were missing data on whether the treatment or other circumstances were the probable cause of that side effect.

The following statements and number of instances were reported as “probably caused by the treatment I received”: “I had more problems with my sleep” (1) “I felt like I was under more stress” (2), “I experienced more anxiety” (1), “I experienced more hopelessness” (1), “I experienced more unpleasant feelings” (3), “Unpleasant memories resurfaced” (4), “I think that I have developed a dependency on my treatment” (1), and “I did not always understand my treatment” (1).

The extent to which the responder felt affected ranged between 0 (“not at all”) and 3 (“very”). Two instances of “very affected” were reported for “I felt like I was under more stress,” one instance was reported under “I experienced more anxiety,” and one instance was reported under “I experienced more unpleasant feelings.” This indicates that RF-CBT provided in a group setting on a comorbid sample may have adverse side effects and incidents.

## 4. Discussion

The results of this RCT suggest that the RF-CBT group intervention is effective for insomnia (in line with the hypothesis) with large (post) and medium (2-month follow-up) effect sizes and potentially for depression with a medium effect size (2-month follow-up, partly in line/partly at odds with the hypothesis). These improvements remained at the 6-month follow-up. Whilst there were also moderate effect sizes for the effect of the RF-CBT group relative to wait-list control group on anxiety and RNT, there were no statistically significant differences (at odds with the hypothesis). Neither were there any significant differences concerning worry, and quality of life (also at odds with the hypothesis).

It is noteworthy that insomnia severity was significantly lower for those who had received RF-CBT compared to the wait-list control group, even though insomnia was not directly addressed in the treatment protocol (i.e., there were no examples or exercises that specifically addressed sleep) and considering the low statistical power to detect small and moderate effects. This finding, however, is consistent with the hypothesis that elements within RF-CBT designed to reduce RNT and avoidance also improve symptoms of insomnia (Harvey et al., 2004; Ehring and Watkins, 2008). Also, RF-CBT did not significantly reduce RNT relative to the wait-list control group, which is at odds with the general hypothesised mechanism of change. The current results may therefore also add some uncertainty to the theoretical link between RNT and insomnia although the power related issues make such inferences difficult. Thus, if these findings are replicated, it will be important to investigate other processes through which RF-CBT may influence insomnia.

Further, depressive symptoms did not differ significantly between the arms at the post-treatment measurement but during the 2-month follow-up. This is also important to note since previous research has demonstrated clear effects of RF-CBT on depressive symptoms (Watkins et al., 2007, 2011; Topper et al., 2017; Hvenegaard et al., 2020). Moving back to insomnia, considering the well-established role of insomnia as a risk factor for depression (Baglioni et al., 2011), the results could be interpreted as if the risk of depression has been lowered in the treatment condition. Perhaps this is why a lower prevalence of depressive symptoms was found at the follow-up, only after a potential maintaining factor (insomnia) had decreased.

There are several potential alternative explanations to the null-results at odds with the hypotheses. First, it cannot be ruled out that the lack of significant time by group effects for RNT, anxiety, and depressive symptoms (post-treatment) are explained by low statistical power to detect small-to-moderate effects, or on significant attrition at the post-and at the two-month follow-up measurements. In other words, the variability in the data in relation to the sample size and the mean differences between the conditions, may have been too high to detect statistically significant effects. In order not to further increase the risk of type 2-errors, no adjustment for multiple testing was made even though it increases the risk of type 1-errors. Second, only 23 out of the 36 (64%) participants randomised into the treatment condition attended >5 sessions, only nine out of 36 (25%) attended all eight sessions, and eight (22%) attended no sessions. This may mean that any genuine treatment effect of the RF-CBT groups would be diluted as a significant proportion of patients received little or no exposure to the RF-CBT treatment. However, a meta-analysis found that the average dropout rates in clinical studies with cognitive behavioural therapy in groups were 14,5% (95% CI = 9.7, 21.0%) at baseline and 24,6% (95% CI = 19.9, 30.1%) during treatment (Fernandez et al., 2015). Thus, the drop-out rates in the current study (attended <6 sessions) were close to the upper Cl for both baseline and during treatment. Third. the intervention dose was smaller than in a comparable study (Møller et al., 2017). The current treatment was administered over a shorter period, with eight instead of 12 sessions at 2 h instead of 3 h per session, and an individual session was not offered before the group sessions. However, group-based RF-CBT have been administered over 6 to 10 sessions, and 6 sessions was found effective for participants with clinical problems (Watkins, 2018). Fourth, a transdiagnostic sample with symptoms within two out of three diagnostic areas was included in the study. Thus, it is not necessarily expected that participants with clinical symptom levels within the areas of anxiety and insomnia but not within depression at baseline would report decreased severity of depressive symptoms (i.e., fewer differences on average between the arms and larger in-group variance as compared to samples with consistent symptomology were expected). This should make it harder to detect differences between the arms.

### 4.1. Limitations

First, the power calculation was based on large effects and significant attrition at the post and follow-up measurements increased the risk of type 2 errors. In order not to further increase the risk of type 2-errors, no adjustment for multiple testing was made even though it increases the risk of type 1-errors.

Second, significant proportions of the participants attended less than six sessions. As previously mentioned, no systematic data on reasons for drop out was collected. However, there are several general potential explanations for the relatively high dropout rate. For example, the clinical contexts will generally suffer from greater attrition (in our case a primary healthcare centre) than more controlled research settings such as a university clinic setting. Also, more health issues (several as opposed to one problem area) might mean greater difficulties tolerating or participating in treatment and a lower willingness to wait for treatment. Since the protocol was addressing RNT rather than problems that are specific to a disorder, it is possible that at least some examples that was used to illustrate problems did not engage the participants enough.

Third, Since PTQ are less evaluated than PSWQ and lacks clinical norms, because of the considerable overlap between the concepts, and since the worry component of RNT was expected to be more prominent than depressive rumination in this heterogenous sample, PSWQ was used to assess clinical levels of worry (thus RNT) during the recruitment in this study. In this context, it is important to note that RF-CBT was originally developed for people with elevated levels of depressive rumination rather than trait worry. According to Spinhoven et al. (2018), most studies on interventions that have addressed RNT in depressive samples have operationalised RNT with the original version of the Ruminative Responses Scale, although PSWQ have also frequently been used. This way of operationalizing RNT may have affected the recruitment in such a way that the sample deviates from other studies with different means for screening for RNT and therefore might make comparisons between treatment effects between trials more difficult.

Future research should aim to address the methodological shortcomings of the current study. It should be highlighted however, that clinical research consists of multiple dilemmas and non-the least concerning maintaining both ecological validity and scientific rigour. But what was lost in one end may allow for gains in another. Fewer participants with more heterogenous symptoms who gets a lower dose of a group-treatment with higher attrition-and drop-out rates better reflect the nature of clinical reality. Although significant attrition indeed may increase the risk for type-1 errors, the risk of type-2 errors would be a lot greater in this study. Considering treatment dose, dropout rates, the sample size (i.e., the standard deviation would be expected to be greater with a smaller sample), the more remarkable it seems that significant improvements of insomnia symptoms were detected. Future research should also consider conducting component analysis of the different elements within RF-CBT (i.e., dismantling).

### 4.2. Strengths

The following strengths of the study should be emphasised. First, the treatment was administered in a naturalistic setting at a primary health care centre on a heterogenous sample in collaboration with therapists who worked there. This strengthens the ecological and external validity.

Second, it was the first study to look at the effects of RF-CBT on a sample with clinical symptom levels within the common areas of depression, anxiety, and insomnia.

Third, the current study was a randomised controlled trial. This has long been considered the gold standard when evaluating treatment effects. Linear mixed models also deal efficiently with missing data and provide unbiased estimates under acceptable assumptions.

Finally, the participants in the waitlist condition did not start their treatment until after the 2-month follow-up, which strengthens internal validity.

## 5. Conclusion

RF-CBT provided in a primary care health care setting and delivered in groups for individuals with clinical symptom levels of at least two disorders appeared effective for insomnia and potentially effective for depression symptomatology. Future research should aim to increase knowledge under which circumstances (when, how and for whom) RF-CBT might be considered the first treatment choice and replicate these findings in a larger definitive sample.

## Data availability statement

The raw data supporting the conclusions of this article will be made available by the authors, without undue reservation.

## Ethics statement

The studies involving human participants were reviewed and approved by The regional ethics review board in Uppsala, Sweden Dnr: 2018/197 and Dnr: 2019-00987. The patients/participants provided their written informed consent to participate in this study.

## Author contributions

MA and OE: funding acquisition, investigation, resources, and writing – review and editing. IH, TK, and VL: investigation and writing – review and editing. HH: data curation, formal Analysis, visualisation, and writing – review and editing. AN: funding acquisition, conceptualization, data curation, investigation, methodology, resources, supervision, visualisation, writing – original draft, and writing – review and editing. MT: funding acquisition, conceptualisation, investigation, methodology, project administration, resources, supervision, visualisation, writing – original draft, and writing – review and editing. DW: data curation, formal analysis, investigation, resources, visualisation, writing – original draft, and writing – review and editing. EW: conceptualisation, methodology, and writing – review and editing. All authors contributed to the article and approved the submitted version.

## Funding

This work was supported by the County Council in Värmland (LIVFOU-763591, 2018–2019).

## Conflict of interest

The authors declare that the research was conducted in the absence of any commercial or financial relationships that could be construed as a potential conflict of interest.

## Publisher’s note

All claims expressed in this article are solely those of the authors and do not necessarily represent those of their affiliated organizations, or those of the publisher, the editors and the reviewers. Any product that may be evaluated in this article, or claim that may be made by its manufacturer, is not guaranteed or endorsed by the publisher.

## Footnotes

^1^In the pre-registration, >45 was mistakenly specified as the clinical threshold.

^2^The threshold was later corrected to ≥34 and an updated approval was obtained from regional ethics review board in Uppsala; Dnr: 2019-00987.

^3^The ongoing 1:1 randomisation of each enrolled pair before the baseline measurements were collected resulted in 7 participants (3 in the treatment-and 4 in the waitlist condition) who were enrolled but never answered the baseline measurements.

^4^Due to logistics, there could be up to 2 weeks between the pre-treatment measurements and first session, thus post-treatment measurements were conducted in close proximity to the treatment ending.

^5^Due to a mistake in the pre-registration process, the two-month follow-up measurements were not specified. Also, mid-measurements that were intended for analyses outside of the main article were specified in the pre-registration but not included in the present analyses.

^6^Since seven participants (three in the treatment- and four in the waitlist condition) were randomised but did not complete the baseline measurements, strict ITT was not maintained.

^7^The ongoing 1:1 block randomization allowed participants to cancel their participation after enrolment but before submitting their baseline measurements.
