# Supplementary material for: Treatment of worry and comorbid symptoms within depression, anxiety, and insomnia with a group-based rumination-focused cognitive-behaviour therapy in a primary health care setting: a randomised controlled trial
Source: Front Psychol. 2023 Sep 7;14:1196945. doi: 10.3389/fpsyg.2023.1196945 (PMC10513770; doi:10.3389/fpsyg.2023.1196945)
Supplement: Supplementary file 1 [file Table_1.DOCX]

Supplementary Material

Treatment of Worry and Comorbid Symptoms within Depression, Anxiety, and Insomnia with a Group-based Rumination-Focused Cognitive-Behaviour Therapy in a Primary Health Care Setting: A Randomised Controlled Trial

**Daniel Wallsten^1*^, Annika Norell^,2,3^, Malin Anniko^3^, Oskar Eriksson^4^, Varja Lamourín^3^, Ida Halldin^3^, Tina Kindbom^3^, Hugo Hesser^3,5^, Edward Watkins^6^, Maria Tillfors^1^**

*** Correspondence:** Daniel Wallsten (daniel.wallsten@kau.se)

# Supplementary Data

**Appendix**

**Table 6.**

*Total number of participants in each condition, number- and percentage of participants within each condition moving across the clinical cut-off levels between the baseline- and post-measurements, between the pre- and 2-month follow-up measurements and between pre- and 6-month follow-up measurements.*

|  |  | n^PRE^ | n^POST^ | n^imp.^ ^PRE-POST^ | % | n^det. PRE-POST^ | % | n^2-m^ | n^imp. PRE-2-m^ | % | n^det. PRE-2-m^ | % | n^6-m^ | n^imp. PRE-6-m^ | % | n^det. PRE-6-m^ | % |
| --- | --- | --- | --- | --- | --- | --- | --- | --- | --- | --- | --- | --- | --- | --- | --- | --- | --- |
| Total n | RFKBT | 33 | 23 |  |  |  |  | 18 |  |  |  |  | 18 |  |  |  |  |
|  | WAITL | 33 | 24 |  |  |  |  | 19 |  |  |  |  | n/a |  |  |  |  |
| PSWQ (≥45) | RFKBT | 31 | 17 | 6 | 19% | 0 | 0% | 12 | 6 | 19% | 0 | 0% | 13 | 6 | 19% | 0 | 0% |
|  | WAITL | 33 | 23 | 1 | 3% | 0 | 0% | 18 | 1 | 3% | 0 | 0% |  |  |  |  |  |
| ISI (≥8) | RFKBT | 31 | 17 | 6 | 19% | 1 | 3% | 11 | 6 | 19% | 0 | 0% | 14 | 4 | 13% | 0 | 0% |
|  | WAITL | 30 | 21 | 2 | 7% | 2 | 7% | 15 | 2 | 7% | 0 | 0% |  |  |  |  |  |
| OASIS (≥8) | RFKBT | 16 | 7 | 3 | 19% | 0 | 0% | 3 | 5 | 31% | 0 | 0% | 3 | 5 | 31% | 0 | 0% |
|  | WAITL | 18 | 12 | 4 | 22% | 3 | 17% | 5 | 4 | 22% | 1 | 6% |  |  |  |  |  |
| MADRS (≥13) | RFKBT | 23 | 13 | 4 | 17% | 1 | 4% | 5 | 7 | 30% | 0 | 0% | 8 | 8 | 35% | 2 | 9% |
|  | WAITL | 31 | 18 | 5 | 16% | 0 | 0% | 15 | 4 | 13% | 1 | 3% |  |  |  |  |  |

*Note.* 2-m = 2-month follow-up, 6-m = 6-month follow-up, imp = improved, det = deteriorated, PSWQ = Penn State Worry Questionnaire, ISI = Insomnia Severity Index; OASIS = Overall Anxiety Severity and Impairment Scale; MADRS-S = Montgomery-Åsberg Depression Rating Scale Self-rated

**Table 7**

*External review of therapist adherence from a randomised selection of sessions*

| Treatment group | 1 | 1 | 2 | 2 | 3 | 3 | 4 | 4 |
| --- | --- | --- | --- | --- | --- | --- | --- | --- |
| Session | 2 | 6 | 3 | 5 | 5 | 7 | 5 | 6 |
| Element |  |  |  |  |  |  |  |  |
| Prev. home assignment | 3 | 3 | 3 | 3 | 2 | 3 | 3 | 3 |
| Element 1 | 2 | 3 | 3 | 3 | 3 | 3 | 3 | 3 |
| Element 2 | 3 | 3 | 3 | 3 | 3 | 3 | 3 | 3 |
| Element 3 | 3 | 2 | 2 | 3 | 3 | 3 | 3 | 2 |
| Element 4 |  | 3 |  | 2 | 2 | 1 | 3 | 3 |
| Element 5 |  | 3 |  | 3 | 3 | 3 | 3 | 3 |
| Element 6 |  | 2 |  |  |  |  |  | 2 |
| Element 7 |  | 3 |  |  |  |  |  | 2 |
| Element 8 |  | 2 |  |  |  |  |  | 2 |
| Home assignment | 3 | 2 | 3 | 2 | 3 | 2 | 3 | 3 |
| Evaluation | 1 | 3 | 1 | 1 | 3 | 1 | 3 | 1 |

*Note.* Prev = previous. 1 = low adherence, 2 = acceptable adherence, 3 indicated high adherence. Each session may contain different amounts of elements.

**Table 8**

*Participant adherence: number, percentage and average of attended sessions among the participants within the treatment condition.*

​​

|  | Session Attendance |  |  |  |
| --- | --- | --- | --- | --- |
| Session | *n* present (%) | *n* absent (%) | *n* dropout (%) |  |
| 1 | 28(78) | 8(22) | 8(22) |  |
| 2 | 26(72) | 10(28) | 0(0) |  |
| 3 | 27(75) | 9(25) | 1(3) |  |
| 4 | 20(56) | 16(44) | 2(6) |  |
| 5 | 22(61) | 14(39) | 0(0) |  |
| 6 | 19(53) | 17(47) | 0(0) |  |
| 7 | 21(58) | 15(42) | 1(3) |  |
| 8 | 20(56) | 16(44) | 0(0) |  |
|  | *M* number of attended sessions (SD) | |  |  |
|  | 5,1(3,1) |  |  |  |

*Note.* dropout = The participant was absent from all remaining sessions including the current.

**Table 9**

*Follow-up attrition within each condition.*

|  | Follow-up attrition |  |  |  |
| --- | --- | --- | --- | --- |
| Group | Measurement | *n* collected (%) | *n* missing (%) | *n* dropout (%) |
| RF-CBT | BASELINE | 33(92) | 3(8) | 3(8) |
|  | POST | 23(64) | 13(36) | 9(25) |
|  | 2-m FU | 18(50) | 18(50) | 2(6) |
|  | 6-m FU | 18(50) | 18(50) | 4(11) |
| Waiting list | BASELINE | 33(89) | 4(11) | 4(11) |
|  | POST | 24(65) | 13(35) | 6(16) |

*Note.* 2-m FU = 2-month follow-up, 6-m FU = 6-month follow-up dropout = all measurements were missing from the given occasion (including the current)

**
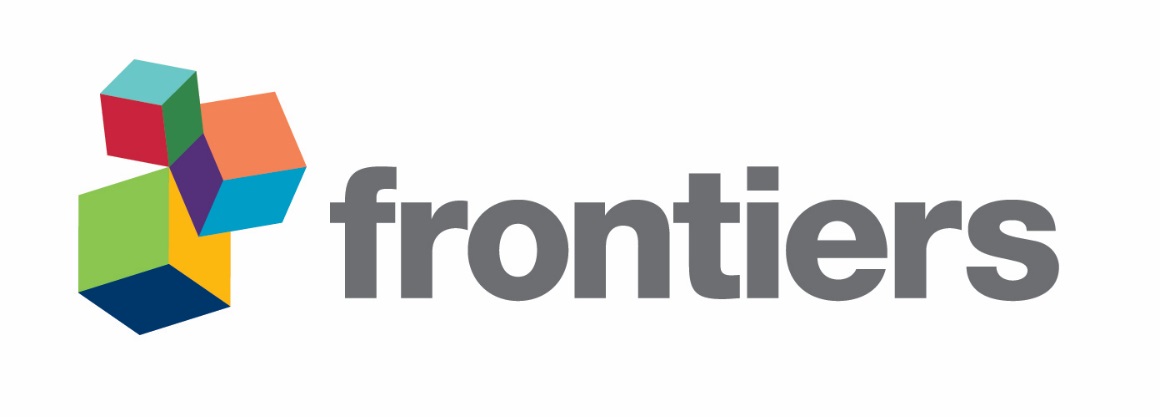
**
